# Testosterone May Hold Therapeutic Promise for the Treatment of Ischemic Stroke in Aging: A Closer Look at Laboratory Findings

**DOI:** 10.15171/apb.2019.006

**Published:** 2018-02-21

**Authors:** Fereshteh Farajdokht, Mehdi Farhoudi, Alireza Majdi, Masumeh Zamanlu, Saeed Sadigh-Eteghad, Shabnam Vahedi, Javad Mahmoudi

**Affiliations:** Neurosciences Research Center (NSRC), Tabriz University of Medical Sciences, Tabriz, Iran.

**Keywords:** Male sex, Testosterone, Stroke, Oxidative stress, Apoptosis

## Abstract

Male sex is more prone to cerebrovascular disorders, yet the exact role of androgens in cerebral ischemia remains unclear. Here we reviewed current understanding of testosterone (TES) neuroprotective activity against ischemic stroke and mechanisms underlying these effects in aging. TES may exert a neuroprotective effect in aging through pathways including inhibition of oxidant molecules production, enhancing the enzymatic antioxidant capacity of the brain and modulation of apoptotic cell death. Given this, a better understanding of the neuroprotective roles of TES may propose an effective therapeutic strategy to improve the quality of life and decrease androgen-related cerebrovascular problems in the aging men.

## Introduction

Stroke is a highly disabling cerebrovascular disease among the elderlies associating with significant mortality and morbidity and considerable economic burden.^1^ It accounts for more than 6 million deaths annually and the number of stroke victims will increase nearly 20% by 2030.^2^ Moreover, annual direct and indirect costs for stroke is estimated to increase more than 2 fold from 2010 to 2030, reaching around 240.67 billion $ by 2030 in the United States.^3^ Hence, it is proposed to become even more crucial health care problem in upcoming years.^4^

Male sex is considered as an important risk factor for stroke. In comparison with age-matched women, the overall incidence of stroke in men is high indicating that sex steroids may have a role in the pathophysiology of stroke.^5^ There is a link between low circulating testosterone (TES) levels and incidence of cerebrovascular events such as transient ischemic attack and ischemic stroke in men. Also, low levels of TES appears to be involved in clinical outcomes of ischemic stroke survivors.^6,7^ Moreover, some of the major stroke risk factors such as cardiovascular disorders,^8^ atherosclerosis^9^ and type 2 diabetes^10^ are usually associated with low TES levels in the old men. Given the role of TES in stroke, this paper aims to focus on the different neuroprotective mechanisms of TES in ischemic stroke.

## Testosterone biology and biosynthesis

TES is a steroidal sex hormone largely producing by Leydig cells^11^ localized in the testicular interstitial.^12^ In addition, a small fraction of TES is released by the zona reticularis of the adrenal glands. However, its production is not limited to the men and in women, both ovaries and the adrenal gland are able to produce small amounts of TES.^13^ TES acts as a pro-hormone in the cerebral tissue and nearly 7% of it can be converted to 5α-dihydrotestosterone (DHT) via the activity of 5α-reductase enzyme.^14,15^ Also a small amount of TEs (about 0.5%) is oxidized to 17β-estradiol by aromatase cytochrome P450 enzyme.^13^ Both these molecules are biologically active and mediate some of the TES roles in relation to neuronal cells.^13,16^ About 98% of circulating TES is bound to sex hormone-binding globulin (SHBG) and albumins; however, only small percentage of TES (0.5%-2%) remains in its unbound form and circulates freely throughout the bloodstream.^17,18^ TES has a high affinity for SHGB and is tightly bound to SHGB which makes SHBG-bound TES unavailable to the most of the tissues for action.^18^ In contrast, since TES exhibits low affinity for binding to albumin, it is loosely bound to it.^17,18^ Thence TES only in albumin-bound and its un-bound (free from) is able to influence the target cells.^18^

In men, SHBG levels increases during aging^17^ which leads to more reduction of free TES (2%-3% per year) when compared with total TES (1.6% per year).^19^ Physiologically, only free TES is able to pass via the blood-brain barrier and reach to the cerebral tissue.^20,21^ Given this, decline in free form of TES impacts on its cerebral levels which may be responsible for appearance of some age-related conditions such as Alzheimer’s disease,^22^ Parkinson’s disease^23^ and cerebrovascular events.^6^

**Figure 1:**
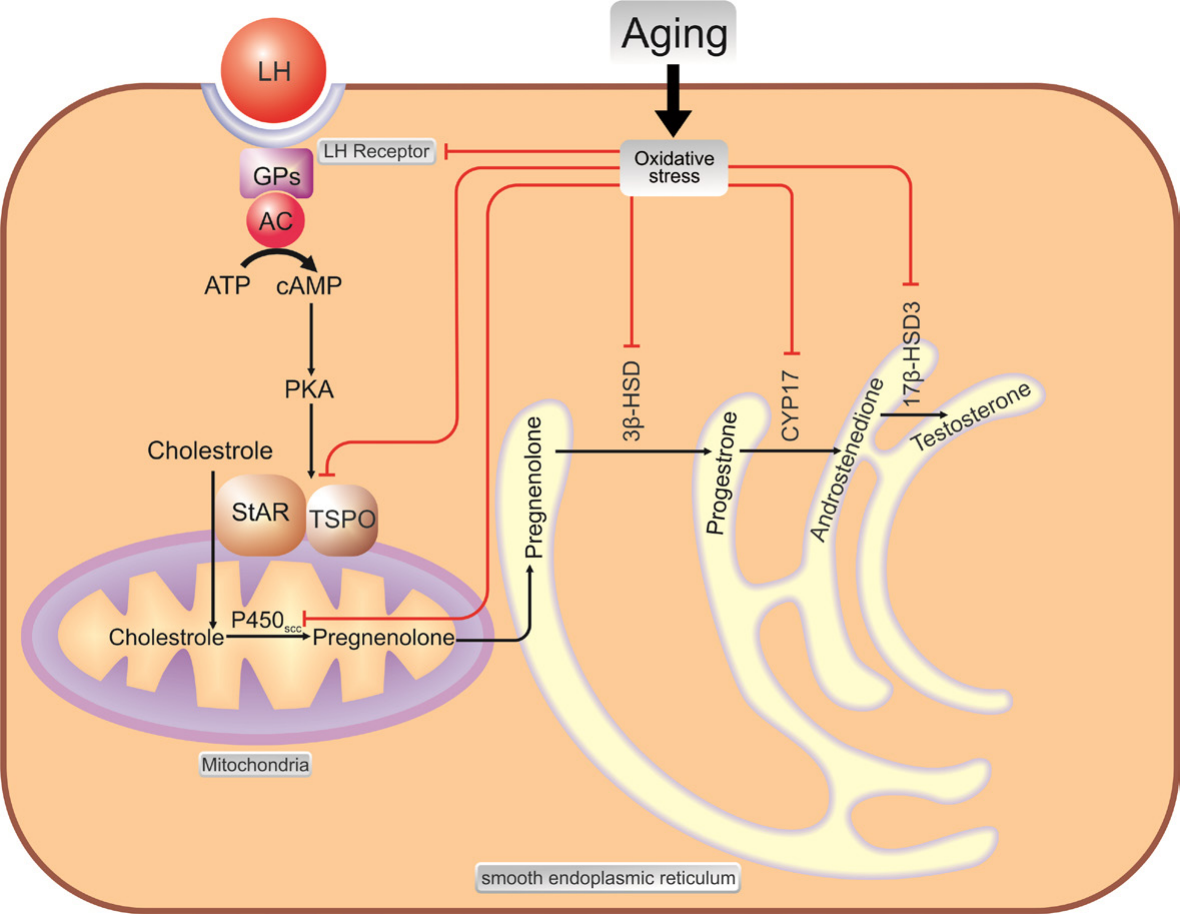
The essential molecular components of testosterone biosynthesis in Leydig cells and the inhibitory effects of aging at multiple levels on these machineries. LH: luteinizing hormone; StAR: steroidogenic acute regulatory protein; TSPO: translocator protein; PKA: protein kinase A; CYP17: 17α-hydroxylase/C17-20 lyase.

Hypothalamic-pituitary system controls gonadal hormones release. Hypothalamus through secretion of gonadotropin-releasing hormone stimulates pituitary gland for releasing of luteinizing hormone (LH). In the testis, LH interacts with its specific receptors and initiates a series of intracellular events for TES biosynthesis.^24^ Stimulation of LH-receptors phosphorylates the steroidogenic acute regulatory protein (StAR) and translocator protein (TSPO),^12,25^ 2 key components for cholesterol trafficking from the cellular pool into the inner mitochondrial membrane.^24-26^ Within the mitochondria, cytochrome P450 enzyme CYP11A1 converts it to pregnenolone.^12^ Then, pregnenolone leaves mitochondria and enters the smooth endoplasmic reticulum, where it changes to progesterone by microsomal 3β-hydroxysteroid dehydrogenase (3β-HSD).^12,25^ Progesterone subsequently underwent oxidation to androstenedione by 17α-hydroxylase/C17-20 lyase (CYP17). Ultimately, androstenedione is metabolized to TES via enzymatic activity of 3-17 β-hydroxysteroid dehydrogenases (17β-HSD3)^12^ (Figure 1).

## Aging and decline in testosterone level

TES deficiency or andropause is characterized with a reduction in total and free TES levels and affects 20%-25% of men above age 65.^6,18,23^ Beside aging,^6^ other conditions such as age-related comorbid disorders and applied medical interventions can also affect TES levels in elderlies.^18^ This state leads to changes in body composition,^27^ insulin resistance,^10^ obesity, reduction of muscle mass, increase of fat mass^28^ as well as sexual and emotional dysfunctions.^18,27,28^ Although there is no comprehensive data about mechanisms underlying TES decline in aged men, evidence shows either number or ability of Leydig cells for production of TES^24,29^ are reduced by 50% in aging.^27^ Given this, it seems that impaired steroidogenic pathway in the aged Leydig cells may have a pivotal role in this condition.^12,30^ Based on oxidative stress theory of aging, long-term oxidative stress happens in aerobic organisms under normal physiologic condition^12^ due to excessive production and deposition of superoxide and other reactive oxygen species (ROS) as well as the disability of cells to clearance of these active molecules.^12,24^ These processes result in oxidative injuries to intracellular biologic macromolecules such as proteins, lipids, and DNA.^24,31,32^

Leydig cells are highly prone to oxidative insults likely due to the production of ROS by mitochondrial electron transport chain and containing P450 enzymes that mediate oxidation of their relevant substrates in the steroidogenic pathway.^24^ Interestingly, macrophages which are resident in the interstitial compartment of testes produce ROS and increased the vulnerability of the Leydig cells toward oxidative damage.^27^ Therefore, these cells are specialized to express a high amount of scavenging molecules such as superoxide dismutase, glutathione peroxidase, and glutathione. However, their capacity to neutralisation of reactive molecules significantly decreased with aging,^24,27^ which this lead to oxidative injury to those essential components of the steroidogenic pathway.^24,33,34^ Findings show that activity of components such as smooth endoplasmic reticulum content (3β-HSD, P450_17_α, and 17β-HSD),^30^ cholesterol transfers; StAR, TSPO and mitochondrial P450scc enzyme^34^ are impaired upon oxidative stress during aging^12,27,34^ (Figure 1). Also, activation of LH receptors and intracellular levels of cAMP in the Leydig cells are necessary for normal function of this well-organized pathway. Therefore, impaired LH-cAMP signaling cascade decreases the capacity of these cells to produce enough TES.^34,35^ According to the radioligand binding studies, the number and affinity of LH binding sites are reduced by 50%–70% in both aged and LH-suppressed Leydig cells.^35^ However, it seems this reduction does not affect TES production. This event can be explained by 2 facts: first, although activation of LH receptors is necessary for activation of LH-cAMP cascade, maximal activation requires only 10% of the total LH receptors^12,36^; and second, though LH-suppressed cells show even more LH binding than aged cells, under LH stimulation they produce more significant levels of TES^12,35^ and cAMP as well.^35^ These reflect that LH signal transduction is severely affected by aging and disability of these cells to maintain cAMP levels in physiologic amount reduces the phosphorylated amount of StAR and TSPO^12^ and resulting in defective translocation of cholesterol toward the steroidogenic enzymes of the Leydig cells. Although the mechanism(s) underlying of impaired LH receptors transduction are poorly understood, findings show that oxidative stress may influence membrane fluidity^37,38^ and decrease the ability of LH-cAMP function.^35^ Table 1 summarises some deficient factors in the steroidogenic pathway of aged Leydig cells.

**Table 1:** Changes in Leydig cells key components involved in TES synthesis during aging

**Component**	**Affected components**	**Analysis technique**	**References**
LH receptor density	Decreased mRNA level	DNA microarray	^39^
StAR protein	Decreased mRNA level	Northern blotting Real-time quantitative PCR Western blotting	^40-42^
	Decreased protein level		
	Decreased activity		
TSPO protein	Reduction in mRNA and protein levels	Northern blotting and Bradford method	^43^
Mitochondrial P450_Scc_	Decreased mRNA levels	Northern blotting	^40,41^
	Decreased protein levels	Western blotting	
3β-HSD, CYP17 & 17β-HSD3	Decreased mRNA levels	Northern blotting	^30^
	Decreased protein levels	Western blotting	

Abbreviations: StAR, steroidogenic acute regulatory protein; TSPO, translocator protein; 3β-HSD, 3β-hydroxysteroid dehydrogenase.

## Neuroprotective role of the androgenic pathway in stroke

Cerebral ischemia is caused by occlusion of cerebral arteries and interruption of cerebral blood flow resulting in cell death and activation of deleterious cascades in perfusion territory of the affected vessels.^2,44^ Till now, two primary strategies have been proposed for remission of ischemic stroke consequences. Firstly vascular approach,^44^ in which thrombolysis with tissue plasminogen activator (tPA) is used as a first-line option.^1,4^ In spite of using this therapy, morbidity of stroke is still high,^4^ indicating the effectiveness of tPA therapy is doubtful. As a matter of fact, tPA therapeutic advantage is time-dependent (its door-to-needle times is <1 hour)^45^ and only is effective in limited numbers of patients. On the other hand, the risk of subsequent intra-cerebral,^46^ peripheral hemorrhage and occurrence of re-occlusion are associated with this therapy.^1^ The second strategy is the use of neuroprotective regimens,^44^ to prevent or to alleviate ischemic injuries.^47^ To our knowledge, the majority of findings related to the therapeutic role of TES originate from the research conducted in middle cerebral artery occlusion (MCAO) model in male rodents.^5,48,49^ This model provides a site-specific and biphasic focal ischemia condition,^50^ which it consists of two distinct phases, including ischemia phase causing cerebral infarct through the cessation of blood flow to MCAO territory^51^ and reperfusion phase that is exploited by removing of the blockade to the restoration of middle cerebral artery (MCA) blood flow.^52,53^ Therefore, MCAO provides a clinically revealed model to resemble human ischemic stroke which occurs by 80% in the territory of MCA and usually followed by recanalization.^52^

Studies show that TES replacement during reperfusion phase of MCAO improves neurochemical, histological^5^ and behavioral outcomes of ischemic strokes in castrated rats.^5,49^ The brain acts as an androgen-responsive organ^54^ in which TES and DHT interact with androgenic receptors (ARs) in order to regulate different neurological functions.^20^ Beside this, 17β-estradiol, an aromatization product of TES, can activate estrogen pathways in the brain.^16,54^ This pathway not only involves in androgenic signaling, but also contributes to neuroprotection procedures.^16^ It has been suggested that both of these mechanisms (activation of ARs and estrogen pathways) reduce the severity of ischemic insults in rodents.^54,55^ Pharmacologic silencing of ARs could improve the neuroprotective effect of TES in the MCAO model possibly via elevation of available TES for metabolization to 17β-estradiol.^54,55^

## Neuroprotective mechanisms of testosterone

### Effects on oxidative stress

**Figure 2:**
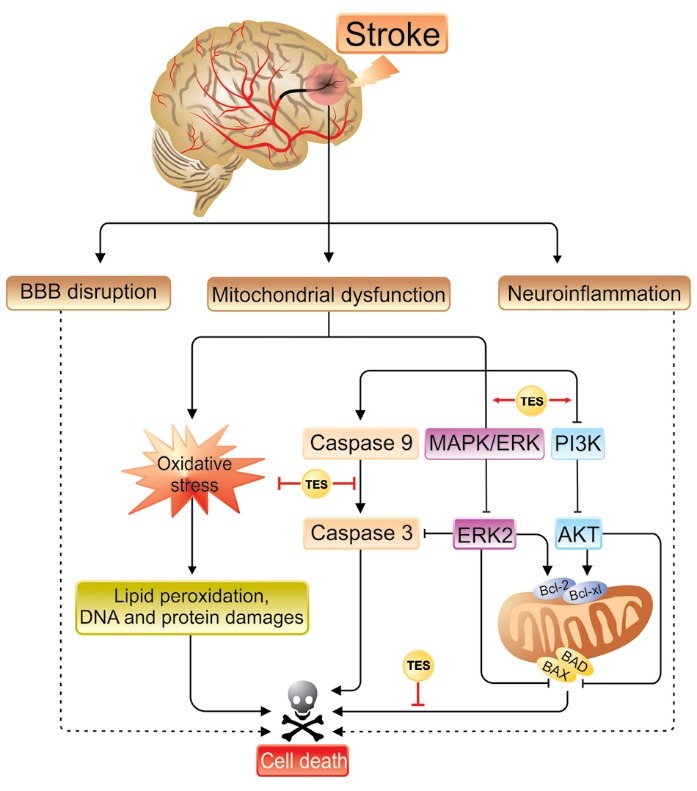
Ischemic stroke triggers cascades of complex events resulting in the neuronal loss in affected area. Testosterone induces neuroprotection in the neuronal cell following cerebral ischemia through inhibition of oxidative stress and blocking apoptotic cell death. ROS: reactive oxygen species; TES: testosterone; MAPK: mitogen-activated protein kinase; ERK: extracellular signal-regulated protein kinase.

Brain tissue is susceptible to oxidative stress damages, due to its high metabolic activity, oxygen consumption and massive levels of peroxidizable lipids.^56^ Moreover, the antioxidant capacity of the brain to neutralize reactive molecules is lower than other tissues.^57,58^ During reperfusion phase of stroke, the excessive amount of O_2_ is delivered to the ischemic neurons to maintain their viability, this impairs mitochondrial respiratory chain via elevation of O_2_ to supra-physiologic levels.^59^ Besides, a deficit in brain antioxidant enzymes could result in macromolecular damages and apoptotic cell death.^5,60,61^ Fanaei et al demonstrated that TES attenuated oxidative stress in mice model of MCAO. According to their findings, post MCAO administration of TES decreases lipid peroxidation and augments superoxide dismutase and catalase activities through activation of ARs.^55^ The anti-oxidant activity of TES is more supported by Túnez et al study. They showed that TES is able to increase cerebral catalase activity and decrease malondialdehyde levels, as a lipid peroxidation marker, in 3-nitropropionic-induced oxidative stress in ovariectomized rats.^62^ In addition, Gürer et al reported increased levels of antioxidant enzymes such as catalase as well as superoxide dismutase and reduced malondialdehyde level following TES administration in a rabbit model of spinal cord ischemic reperfusion injury.^20^ Hence, TES may ischemic injuries and exhibit neuroprotective effect through its antioxidant properties (Figure 2).

### Effects on apoptotic cell death

Necrosis and apoptosis are proposed to be involved in cell death following stroke. Immediately after ischemic stroke, the central core of impacted area undergoes necrotic cell death. This core is surrounded by a moderately hypoperfused penumbra zone that maintains structural integrity.^63^ Penumbra or periinfarct zone comprises nearly half of the total lesion volume during the initial stages of stroke which represent that the area may be recovered by early re-occlusion.^59^

The brain blood flow is 55 mL/100 g/min under the physiologic condition but in penumbra zone, it declines below 18 mL/100 g/min.^64^ Although neurons in the penumbra zone are functionally inactive, their metabolic functions are sustained^2^ and the majority of neurons in this region commit suicide by activating an apoptotic cell death program after the stroke attack.^65^ Contrary to necrotic cell death, apoptosis is a relatively ordered process and is activated by a sequence of biochemical cascades and culminates in energy-dependent programmed cell loss,^65,66^ the cytoplasmic and nuclear condensation, and DNA break into nucleosomal fragments.^67,68^ Apoptosis resulting in the disposal of shrunken remnants of dismantled cells by macrophages without inflammation in order to minimize ischemic injuries to adjacent cells.^66^ Two general pathways of apoptosis, the extrinsic and intrinsic pathways, are activated following stroke in the penumbra.^65^ These pathways depend on the related activity of caspase and Bcl-2 family proteins, consisting of anti-apoptotic (Bcl-2 and Bcl-x) and pro-apoptotic (Bax) members.^69^ Caspases belong to cysteine protease family and supposed to have a role in ischemic reperfusion injuries.^20^ A rapid increase in caspase activity in penumbra zone following reperfusion reflects that inhibition of caspase may have a role to minimize focal ischemic injuries.^63^

A few studies have reported that TES inhibits apoptotic cell death in the experimental model of stroke. Persky et al showed that five consecutive days exposure of neonatal rats to exogenous TES decreases their sensitivity to MCAO injuries. They proposed that postnatal administration of TES (but not DHT) enhances circulating estradiol level which ultimately induces the expression of X-linked inhibitor of apoptosis resulting in blockade of activated caspase.^48^ The anti-apoptotic role of TES is more supported by Gürer et al showing TES administration in part through caspase-3 inhibition reduces apoptosis in ischemia/reperfusion spinal cord injuries and improves functional recovery.^20^ The neuroprotective effect of androgens in cerebral ischemia is also associated with PI3K**/**Akt signaling pathway.^54^ Following ischemia, this pathway is activated and regulates apoptotic cell death through up-regulation of anti-apoptotic members such as Bcl-2 and Bcl-xL, which improves neuronal survival.^70^ Moreover, androgens through an AR-dependent signaling cascade stimulate mitogen-activated protein kinase/extracellular signal-regulated protein kinase (MAPK/ERK) pathway, followed by inhibiting of phosphorylation of the pro-apoptotic protein Bad, resulting in decreasing apoptotic cell death^71^ (Figure 2).

### Effects on brain neuronal integrity

Aging also influences the integrity of the neurovascular unit which may accelerate brain injury following ischemic stroke.^72^ Cellular and molecular studies also show protective effects of TES on brain microvasculature, and physiological levels of TES and its metabolite diminish infarct damage after MACAO in castrated mice.^20,71,73^ Effects of TES levels and replacement on neuronal structure, blood-brain barrier (BBB), and neuroinflammation have been studied in rodents, though not in the context of brain injury. A study shows that age-dependent variations in TES levels are a causative issue to age-associated white matter impairment. Beilecky et al reported that TES and its receptor have a central role in the myelin regeneration in mice. They showed that TES and ARs are involved in the astrocyte recruitment into a demyelinated lesion and spontaneous oligodendrocyte-mediated remyelination. However, in the absence of testes, TES, as well as ARs remyelination is markedly repressed in castrated mice.^74^ Stroke also results in disruption of the BBB and increases its permeability and the entry of immune cells.^75,76^ So far, limited data are available in the literature about the role of TES on BBB structure. Barreto et al. reported that TES reduces reactive astroglia and reactive microglia after brain injury in gonadectomized male rats.^77^ A recent study also demonstrated that TES depletion is associated with BBB permeability, activation of astroglia and microglia, and up-regulation of inflammatory mediators in the medial preoptic area. Nevertheless, TES replacement for 30 days restored BBB permeability, tight junction integrity and attenuated inflammation in castrated male mice.^78^ Moreover, in vitro study showed that TES up-regulates aquaporin-4, an astrocyte-specific water channel, expression in the cultured astrocytes concomitantly with a decrease in astrocyte osmotic fragility indicating a protective effect of TES against brain edema.^79^

Furthermore, dysfunction of the immune system is the main contributor to morbidity and long-term recovery following ischemic stroke.^80^ Therefore, it is important to examine the influence of TES levels on immune function following stroke. Human studies showed that elderly men with hypogonadism have high serum TNF-α and IL-6 levels, and TES therapy attenuates pro-inflammatory cytokines and increases anti-inflammatory mediators such as IL-10.^81-83^

## The possible health risks of TES replacement therapy

There is controversy regarding indications of TES supplementation in aging men. In spite of this controversy, TES supplementation in the United States has increased considerably over the past several years and the US Food and Drug Administration (FDA) has warned that exogenous TES supplementation is approved only for men who have low TES levels to restore its levels at as close to physiologic concentrations.^84,85^

Evidence shows that restoring TES levels to within the normal range in aging men with hypogonadism produce a wide range of benefits including improvement in sexual function and libido, body composition, bone density and muscle mass, mood, cognition, and cardiovascular disease.^86^ While TES replacement has several benefits which enhance the quality of life of patients, there is some evidence on the risks of TES use.^86,87^ Many of the health risks of TES replacement therapy depend on age, medical conditions, and life circumstances.^88^ Therefore, all elderly men with subnormal TES levels (serum total TES levels < 300 ng/dL), who need TES therapy, should be informed of all risks. TES replacement has been linked to congestive heart failure, benign prostatic hyperplasia, male breast cancer, polycythemia, obstructive sleep apnea, hepatic tumors, hepatotoxicity, and liver failure.^41,89^ Another potential health risks of TES supplementation therapy is stimulation of prostate cancer and benign prostatic hyperplasia, even though there is no conclusive evidence to support this risk.^90-94^ Unfortunately, data on the safety of TES therapy in the aging population is not currently available and large-scale prospective studies addressing the long-term effect of TES and assessing its benefits and risks are needed.

## Conclusion

Based on above, TES neuroprotection against stroke in aging appears to be mediated by several mechanisms including inhibition of production of oxidant molecules, enhancing the enzymatic antioxidant capacity of the brain, activation of PI3K/AKT pathway and enhancing cell survival, inhibition of pro-apoptotic protein through AR-dependent MAPK/ERK pathway, as well as improvement of brain neuronal and BBB integrities. These mechanisms may propose future therapeutic strategies to improve the quality of life and decrease androgen-related health problems in the aging population.

## Ethical Issues

Not applicable.

## Conflict of Interest

All authors declare they have no conflict of interest.

## References

[1] Farhoudi M, Mehrvar K, Sadigh-Eteghad S, Majdi A, Mahmoudi J (2014). A review on molecular mechanisms of reocclusion following thrombolytic therapy in ischemic stroke patients. J Exp Clin Neurosci.

[2] Shaafi S, Mahmoudi J, Pashapour A, Farhoudi M, Sadigh-Eteghad S, Akbari H (2014). Ketogenic diet provides neuroprotective effects against ischemic stroke neuronal damages. Adv Pharm Bull.

[3] Ovbiagele B, Goldstein LB, Higashida RT, Howard VJ, Johnston SC, Khavjou OA (2013). Forecasting the future of stroke in the United States: a policy statement from the American Heart Association and American Stroke Association. Stroke.

[4] Bagherpour R, Dykstra DD, Barrett AM, Luft AR, Divani AA (2014). A comprehensive neurorehabilitation program should be an integral part of a comprehensive stroke center. Front Neurol.

[5] Fanaei H, Karimian SM, Sadeghipour HR, Hassanzade G, Kasaeian A, Attari F (2014). Testosterone enhances functional recovery after stroke through promotion of antioxidant defenses, BDNF levels and neurogenesis in male rats. Brain Res.

[6] Yeap BB, Hyde Z, Almeida OP, Norman PE, Chubb SP, Jamrozik K (2009). Lower testosterone levels predict incident stroke and transient ischemic attack in older men. J Clin Endocrinol Metab.

[7] Herson PS, Koerner IP, Hurn PD (2009). Sex, sex steroids, and brain injury. Semin Reprod Med.

[8] Zhao SP, Li XP (1998). The association of low plasma testosterone level with coronary artery disease in Chinese men. Int J Cardiol.

[9] Jones RD, Nettleship JE, Kapoor D, Jones HT, Channer KS (2005). Testosterone and atherosclerosis in aging men. Am J Cardiovasc Drugs.

[10] Stellato R, Feldman H, Hamdy O, Horton E, McKinlay JB (2000). Testosterone, sex hormone-binding globulin, and the development of type 2 diabetes in middle-aged men: prospective results from the Massachusetts male aging study. Diabetes Care.

[11] Shima Y, Miyabayashi K, Haraguchi S, Arakawa T, Otake H, Baba T (2012). Contribution of Leydig and Sertoli cells to testosterone production in mouse fetal testes. Mol Endocrinol.

[12] Midzak AS, Chen H, Papadopoulos V, Zirkin BR (2009). Leydig cell aging and the mechanisms of reduced testosterone synthesis. Mol Cell Endocrinol.

[13] Lopes RAM, Neves KB, Carneiro FS, Tostes R (2012). Testosterone and vascular function in aging. Front Physiol.

[14] Thigpen AE, Silver RI, Guileyardo JM, Casey ML, McConnell JD, Russell DW (1993). Tissue distribution and ontogeny of steroid 5 alpha-reductase isozyme expression. J Clin Invest.

[15] Askew EB, Gampe RT, Stanley TB, Faggart JL, Wilson EM (2007). Modulation of androgen receptor activation function 2 by testosterone and dihydrotestosterone. J Biol Chem.

[16] Cheng J, Hu W, Toung TJ, Zhang Z, Parker SM, Roselli CE (2009). Age-dependent effects of testosterone in experimental stroke. J Cereb Blood Flow Metab.

[17] Bialek M, Zaremba P, Borowicz KK, Czuczwar SJ (2004). Neuroprotective role of testosterone in the nervous system. Pol J Pharmacol.

[18] Matsumoto AM (2002). Andropause clinical implications of the decline in serum testosterone levels with aging in men. J Gerontol A Biol Sci Med Sci.

[19] Feldman HA, Longcope C, Derby CA, Johannes CB, Araujo AB, Coviello AD (2002). Age trends in the level of serum testosterone and other hormones in middle-aged men: longitudinal results from the Massachusetts male aging study. J Clin Endocrinol Metab.

[20] Gürer B, Kertmen H, Kasim E, Yilmaz ER, Kanat BH, Sargon MF (2015). Neuroprotective effects of testosterone on ischemia/reperfusion injury of the rabbit spinal cord. Injury.

[21] Pardridge WM, Mietus LJ (1979). Transport of steroid hormones through the rat blood-brain barrier Primary role of albumin-bound hormone. J Clin Invest.

[22] Chu LW, Tam S, Wong RL, Yik PY, Song Y, Cheung BM (2010). Bioavailable testosterone predicts a lower risk of Alzheimer’s disease in older men. J Alzheimers Dis.

[23] Majidi Zolbanin N, Zolali E, Mohajjel Nayebi A (2014). Testosterone Replacement Attenuates Haloperidol-Induced Catalepsy in Male Rats. Adv Pharmac Bull.

[24] Beattie MC, Chen H, Fan J, Papadopoulos V, Miller P, Zirkin BR (2013). Aging and luteinizing hormone effects on reactive oxygen species production and DNA damage in rat Leydig cells. Biol Reprod.

[25] Gomez-Sanchez CE, Qi X, Velarde-Miranda C, Plonczynski MW, Parker CR, Rainey W (2014). Development of monoclonal antibodies against human CYP11B1 and CYP11B2. Mol Cell Endocrinol.

[26] Rone MB, Fan J, Papadopoulos V (2009). Cholesterol transport in steroid biosynthesis: role of protein-protein interactions and implications in disease states. Biochim Biophys Acta.

[27] Zirkin BR, Tenover JL (2012). Aging and declining testosterone: past, present, and hopes for the future. J Androl.

[28] Laughlin GA, Barrett-Connor E, Bergstrom J (2008). Low serum testosterone and mortality in older men. J Clin Endocrinol Metab.

[29] Haji M, Tanaka S, Nishi Y, Yanase T, Takayanagi R, Hasegawa Y (1994). Sertoli cell function declines earlier than Leydig cell function in aging Japanese men. Maturitas.

[30] Luo L, Chen H, Zirkin BR (1996). Are Leydig cell steroidogenic enzymes differentially regulated with aging?. J Androl.

[31] Ames BN, Shigenaga MK, Hagen TM (1993). Oxidants, antioxidants, and the degenerative diseases of aging. Proc Natl Acad Sci U S A.

[32] Khorrami A, Ghanbarzadeh S, Mahmoudi J, Nayebi A, Maleki-Dizaji N, Garjani A (2015). Investigation of the memory impairment in rats fed with oxidized-cholesterol-rich diet employing passive avoidance test. Drug Res (Stuttg).

[33] Chen H, Zirkin BR (1999). Long-term suppression of Leydig cell steroidogenesis prevents Leydig cell aging. Proc Natl Acad Sci U S A.

[34] Zirkin BR, Chen H (2000). Regulation of Leydig cell steroidogenic function during aging. Biol Reprod.

[35] Chen H, Hardy MP, Zirkin BR (2002). Age-related decreases in Leydig cell testosterone production are not restored by exposure to LH in vitro. Endocrinology.

[36] Hsueh A, Dufau M, Catt K (1977). Gonadotropin-induced regulation of luteinizing hormone receptors and desensitization of testicular 3’: 5’-cyclic AMP and testosterone responses. Proc Natl Acad Sci U S A.

[37] Karbownik M, Garcia JJ, Lewiński A, Reiter RJ (2001). Carcinogen-induced, free radical-mediated reduction in microsomal membrane fluidity: reversal by indole-3-propionic acid. J Bioenerg Biomembr.

[38] Vlasova I (2000). The effect of oxidatively modified low-density lipoproteins on platelet aggregability and membrane fluidity. Platelets.

[39] Chen H, Irizarry RA, Luo L, Zirkin BR (2004). Leydig cell gene expression: effects of age and caloric restriction. Exp Gerontol.

[40] Luo L, Chen H, Zirkin BR (2001). Leydig cell aging: steroidogenic acute regulatory protein (StAR) and cholesterol side‐chain cleavage enzyme. J Androl.

[41] Luo L, Chen H, Zirkin BR (2005). Temporal relationships among testosterone production, steroidogenic acute regulatory protein (StAR), and P450 side‐chain cleavage enzyme (P450scc) during Leydig cell aging. J Androl.

[42] Sun Z, Shen W-J, Sucheta S-L, Azhar S (2008). Impact of aging on cholesterol transport protein expression and steroidogenesis in rat testicular Leydig cells. Open Longev Sci.

[43] Culty M, Luo L, Yao ZX, Chen H, Papadopoulos V, Zirkin BR (2002). Cholesterol transport, peripheral benzodiazepine receptor, and steroidogenesis in aging Leydig cells. J Androl.

[44] Siesjö BK (1992). Pathophysiology and treatment of focal cerebral ischemia: Part I: Pathophysiology. J Neurosurg.

[45] Fonarow GC, Zhao X, Smith EE, Saver JL, Reeves MJ, Bhatt DL (2014). Door-to-needle times for tissue plasminogen activator administration and clinical outcomes in acute ischemic stroke before and after a quality improvement initiative. JAMA.

[46] Maeda M, Furuichi Y, Ueyama N, Moriguchi A, Satoh N, Matsuoka N (2002). A combined treatment with tacrolimus (FK506) and recombinant tissue plasminogen activator for thrombotic focal cerebral ischemia in rats[colon] increased neuroprotective efficacy and extended therapeutic time window. J Cereb Blood Flow Metab.

[47] Chen J, Venkat P, Zacharek A, Chopp M (2014). Neurorestorative therapy for stroke. Front Hum Neurosci.

[48] Persky RW, Liu F, Xu Y, Weston G, Levy S, Roselli CE (2013). Neonatal testosterone exposure protects adult male rats from stroke. Neuroendocrinology.

[49] Pan Y, Zhang H, Acharya AB, Patrick PH, Oliver D, Morley JE (2005). Effect of testosterone on functional recovery in a castrate male rat stroke model. Brain Res.

[50] Bachour SP, Hevesi M, Bachour O, Sweis BM, Mahmoudi J, Brekke JA (2016). Comparisons between Garcia, Modo, and Longa rodent stroke scales: Optimizing resource allocation in rat models of focal middle cerebral artery occlusion. J Neurol Sci.

[51] Panahpour H, Dehghani GA (2012). Attenuation of focal cerebral ischemic injury following post-ischemic inhibition of angiotensin converting enzyme (ACE) activity in normotensive rat. Iran Biomed J.

[52] Chiang T, Messing RO, Chou W-H (2011). Mouse model of middle cerebral artery occlusion. J Vis Exp.

[53] Panahpour H, Nekooeian AA, Dehghani GA (2014). Candesartan attenuates ischemic brain edema and protects the blood-brain barrier integrity from ischemia/reperfusion injury in rats. Iran Biomed J.

[54] Liu M, Kelley MH, Herson PS, Hurn PD (2010). Neuroprotection of sex steroids. Minerva Endocrinol.

[55] Fanaei H, Sadeghipour HR, Karimian SM, Hassanzade G (2013). Flutamide enhances neuroprotective effects of testosterone during experimental cerebral ischemia in male rats. ISRN Neurol.

[56] Dringen R (2000). Metabolism and functions of glutathione in brain. Prog Neurobiol.

[57] El Kossi MMH, Zakhary MM (2000). Oxidative stress in the context of acute cerebrovascular stroke. Stroke.

[58] Allen C, Bayraktutan U (2009). Oxidative stress and its role in the pathogenesis of ischaemic stroke. Int J Stroke.

[59] Bretón RR, Rodríguez JCG (2012). Excitotoxicity and oxidative stress in acute ischemic stroke. Stroke.

[60] Panahpour H, Nekooeian AA, Dehghani GA (2014). Blockade of central angiotensin II AT1 receptor protects the brain from ischemia/reperfusion injury in normotensive rats. Iran J Med Sci.

[61] Majdi A, Mahmoudi J, Sadigh‐Eteghad S, Golzari SE, Sabermarouf B, Reyhani‐Rad S (2016). Permissive role of cytosolic pH acidification in neurodegeneration: a closer look at its causes and consequences. J Neurosci Res.

[62] Túnez I, Feijóo M, Collado JA, Medina FJ, Peña J, Muñoz MC (2007). Effect of testosterone on oxidative stress and cell damage induced by 3-nitropropionic acid in striatum of ovariectomized rats. Life Sci.

[63] Yuan J (2009). Neuroprotective strategies targeting apoptotic and necrotic cell death for stroke. Apoptosis.

[64] Bradley WG. Neurology in Clinical Practice: Principles of Diagnosis and Management. Taylor & Francis; 2004.

[65] Broughton BR, Reutens DC, Sobey CG (2009). Apoptotic mechanisms after cerebral ischemia. Stroke.

[66] Woodruff TM, Thundyil J, Tang S-C, Sobey CG, Taylor SM, Arumugam TV (2011). Pathophysiology, treatment, and animal and cellular models of human ischemic stroke. Mol Neurodegener.

[67] Liu X, Zou H, Slaughter C, Wang X (1997). DFF, a heterodimeric protein that functions downstream of caspase-3 to trigger DNA fragmentation during apoptosis. Cell.

[68] Gronbeck KR, Rodrigues CM, Mahmoudi J, Bershad EM, Ling G, Bachour SP (2016). Application of tauroursodeoxycholic acid for treatment of neurological and non-neurological diseases: is there a potential for treating traumatic brain injury?. Neurocrit Care.

[69] Hongmei Z. Extrinsic and Intrinsic Apoptosis Signal Pathway Review. InTech Open; 2012.

[70] Lan R, Xiang J, Zhang Y, Wang G-H, Bao J, Li W-W (2013). PI3K/Akt pathway contributes to neurovascular unit protection of Xiao-Xu-Ming decoction against focal cerebral ischemia and reperfusion injury in rats. Evid Based Complement Alternat Med.

[71] Nguyen TVV, Yao M, Pike CJ (2005). Androgens activate mitogen‐activated protein kinase signaling: Role in neuroprotection. J Neurochem.

[72] Cai W, Zhang K, Li P, Zhu L, Xu J, Yang B (2017). Dysfunction of the neurovascular unit in ischemic stroke and neurodegenerative diseases: An aging effect. Ageing Res Rev.

[73] Uchida M, Palmateer JM, Herson PS, DeVries AC, Cheng J, Hurn PD (2009). Dose-dependent effects of androgens on outcome after focal cerebral ischemia in adult male mice. J Cereb Blood Flow Metab.

[74] Bielecki B, Mattern C, Ghoumari AM, Javaid S, Smietanka K, Abi Ghanem C (2016). Unexpected central role of the androgen receptor in the spontaneous regeneration of myelin. Proc Natl Acad Sci U S A.

[75] Santos Samary C, Pelosi P, Leme Silva P, Rieken Macedo Rocco P (2016). Immunomodulation after ischemic stroke: potential mechanisms and implications for therapy. Crit Care.

[76] Iadecola C, Anrather J (2011). The immunology of stroke: from mechanisms to translation. Nat Med.

[77] Barreto G, Veiga S, Azcoitia I, Garcia‐Segura LM, Garcia‐Ovejero D (2007). Testosterone decreases reactive astroglia and reactive microglia after brain injury in male rats: role of its metabolites, oestradiol and dihydrotestosterone. Eur J Neurosci.

[78] Atallah A, Mhaouty-Kodja S, Grange-Messent V (2017). Chronic depletion of gonadal testosterone leads to blood–brain barrier dysfunction and inflammation in male mice. J Cereb Blood Flow Metab.

[79] Gu F, Hata R, Toku K, Yang L, Ma YJ, Maeda N (2003). Testosterone up‐regulates aquaporin‐4 expression in cultured astrocytes. J Neurosci Res.

[80] Shim R, Wong CHY (2016). Ischemia, Immunosuppression and Infection—Tackling the Predicaments of Post-Stroke Complications. Int J Mol Sci.

[81] Malkin CJ, Pugh PJ, Jones RD, Kapoor D, Channer KS, Jones TH (2004). The effect of testosterone replacement on endogenous inflammatory cytokines and lipid profiles in hypogonadal men. J Clin Endocrinol Metab.

[82] Olsen NJ, Kovacs WJ (1995). Case report: testosterone treatment of systemic lupus erythematosus in a patient with Klinefelter’s syndrome. Am J Med Sci.

[83] Kapoor D, Clarke S, Stanworth R, Channer K, Jones T (2007). The effect of testosterone replacement therapy on adipocytokines and C-reactive protein in hypogonadal men with type 2 diabetes. Eur J Endocrinol.

[84] Food and Drug Administration. FDA Drug Safety Communications: FDA cautions about using testosterone products for low testosterone due to aging; requires labeling change to inform of possible increased risk of heart attack and stroke with use. FDA; 2014. 10.1016/j.juro.2015.06.058 26292880

[85] Nguyen CP, Hirsch MS, Moeny D, Kaul S, Mohamoud M, Joffe HV (2015). Testosterone and “Age-Related Hypogonadism”--FDA Concerns. N Engl J Med.

[86] Bassil N, Alkaade S, Morley JE (2009). The benefits and risks of testosterone replacement therapy: a review. Ther Clin Risk Manag.

[87] Osterberg EC, Bernie AM, Ramasamy R (2014). Risks of testosterone replacement therapy in men. Indian J Urol.

[88] Bhasin S, Cunningham GR, Hayes FJ, Matsumoto AM, Snyder PJ, Swerdloff RS (2006). Testosterone therapy in adult men with androgen deficiency syndromes: an endocrine society clinical practice guideline. J Clin Endocrinol Metab.

[89] Westaby D, Paradinas F, Ogle S, Randell J, Murray-Lyon I (1977). Liver damage from long-term methyltestosterone. The Lancet.

[90] Marks LS, Mazer NA, Mostaghel E, Hess DL, Dorey FJ, Epstein JI (2006). Effect of testosterone replacement therapy on prostate tissue in men with late-onset hypogonadism: a randomized controlled trial. JAMA.

[91] Holyoak JD, Crawford ED, Meacham RB (2008). Testosterone and the prostate: implications for the treatment of hypogonadal men. Curr Urol Rep.

[92] Hormones E, Group PCC (2008). Endogenous sex hormones and prostate cancer: a collaborative analysis of 18 prospective studies. J Natl Cancer Inst.

[93] Wang C, Nieschlag E, Swerdloff R, Behre HM, Hellstrom WJ, Gooren LJ (2009). Investigation, treatment, and monitoring of late‐onset hypogonadism in males: ISA, ISSAM, EAU, EAA, and ASA recommendations. J Androl.

[94] Carpenter WR, Robinson WR, Godley PA (2008). Getting over testosterone: postulating a fresh start for etiologic studies of prostate cancer. J Natl Cancer Inst.

